# Comfort-Oriented Pothole Traversal Using Multi-Sensor Perception and Fuzzy Control

**DOI:** 10.3390/s26061925

**Published:** 2026-03-19

**Authors:** Chaochun Yuan, Shiqi Hang, Youguo He, Jie Shen, Long Chen, Yingfeng Cai, Shuofeng Weng, Junxian Wang

**Affiliations:** 1 Automotive Engineering Research Institute, Jiangsu University, Zhenjiang 212013, China; hangsq_01@163.com (S.H.); hyg197715@163.com (Y.H.); chenlong@ujs.edu.cn (L.C.); caicaixiao0304@126.com (Y.C.); wengshuof@163.com (S.W.); 2 Department of Computer and Information Science, University of Michigan-Dearborn, Dearborn, MI 48128, USA; shen@umich.edu; 3 School of Automotive Engineering, Wuxi Institute of Communications Technology, Wuxi 214100, China; wangjunxian2021@163.com

**Keywords:** intelligent vehicle, pothole traversal, pothole detection, image processing, driving comfort

## Abstract

Potholes are typical negative road obstacles that can significantly compromise vehicle safety and ride comfort when traversed at inappropriate speeds. To address this issue, this paper proposes a pothole-detection-based, comfort-oriented pothole traversal algorithm that integrates multi-sensor fusion perception, comfort-constrained speed planning, and fuzzy control. A camera and a single-point ranging LiDAR are first fused to extract key geometric features of potholes, including contour, area, and depth. Based on these features, a vehicle–pothole dynamic model is developed in ADAMS to quantify the influence of pothole area and depth on vehicle vertical vibration. The vertical frequency-weighted root-mean-square (RMS) acceleration is adopted as the ride comfort indicator, based on which the maximum allowable traversal speed under different pothole geometries is determined. Furthermore, a longitudinal pothole traversal control strategy based on fuzzy theory is designed to regulate vehicle acceleration, enabling the vehicle to reach the comfort-constrained limiting speed within a finite preview distance while ensuring braking safety. The proposed method is validated through multi-scenario co-simulations using MATLAB/Simulink and CarSim, as well as real-vehicle experiments. Results demonstrate that the proposed strategy can effectively adjust vehicle speed before pothole traversal, satisfying comfort constraints and improving ride comfort without sacrificing driving safety.

## 1. Introduction

Road potholes, as typical negative road obstacles, are widely distributed on urban roads and rural highways. When a vehicle traverses a pothole at an inappropriate speed, severe vertical impacts and vibrations may be induced, leading to degraded ride comfort, accelerated damage to suspension and tires, and, under high-speed or emergency conditions, an increased risk of vehicle instability, thereby posing significant threats to driving safety [1]. With the rapid development of advanced driver-assistance systems (ADASs), vehicles have gained enhanced capabilities for road and environment perception. Detecting potholes and timely regulating vehicle speed during pothole traversal have thus become important research directions for improving both driving safety and ride comfort. However, most existing studies primarily focus on pothole detection, while relatively limited attention has been paid to analyzing the vehicle dynamic effects induced by potholes and developing corresponding traversal strategies. This gap restricts the transformation of pothole perception results into executable vehicle control actions.

Active collision avoidance systems can assess driving risks based on real-time traffic environments and ego-vehicle states, and subsequently take appropriate measures to mitigate risks and improve vehicle safety [2,3]. Most active collision avoidance control algorithms concentrate on collision risk avoidance and stability control with respect to vehicles, pedestrians, or other obstacles. In this study, active vehicle collision avoidance concepts are integrated with pothole detection to enhance safety and ride comfort during pothole traversal, where “pothole traversal” refers to safely passing over a pothole through comfort-oriented speed planning and control rather than bypassing it via lateral maneuvers.

Pothole detection methods can generally be classified into two categories: contact-based and non-contact-based approaches, depending on whether the vehicle needs to physically pass over the pothole, and these methods have gradually been integrated with intelligent driving decision-making and control systems [4]. Contact-based methods mainly rely on external or onboard sensors, such as accelerometers and gyroscopes, to collect vehicle vibration responses for detecting road anomalies, including potholes [5]. Xue et al. [6] utilized smartphone-embedded vibration sensors together with a single-degree-of-freedom vehicle vibration model to infer pothole depth and length. Özoğlu et al. [7] further incorporated GPS information to construct a road anomaly detection system capable of identifying pothole events. These approaches are characterized by low cost and ease of deployment; however, detection occurs only after the vehicle has already traversed the pothole, making it difficult to provide effective preview information. Moreover, vibration-based signals are highly sensitive to variations in vehicle type, load conditions, and suspension characteristics, which may result in false detections and limit their general applicability across different vehicle platforms.

Non-contact pothole detection methods primarily utilize onboard cameras, LiDARs, and other sensors to acquire planar and three-dimensional information, employing vision-based or multi-sensor fusion techniques for pothole identification. In recent years, object detection algorithms such as YOLO, as well as convolutional neural networks (CNNs), have been widely applied to pothole image recognition [8,9,10,11]. Ruseruka et al. [12] employed a YOLO-based framework to detect potholes and estimate their dimensions, improving road inspection and maintenance efficiency. Satti et al. [13] introduced cascade classifiers and vision transformers to enhance feature extraction performance in complex environments involving blind spots, water-filled potholes, and illuminated traffic signs. Fan et al. [14] proposed a pothole detection method based on road disparity map estimation and segmentation, using extended perspective transformation and semi-global matching to compute road disparities, followed by superpixel-based geometric detection. Guan et al. [15] developed a pothole detection system combining binocular vision and deep learning to improve forward detection accuracy. Despite their preview advantages, vision-based approaches alone struggle to reliably estimate pothole depth and damage severity from two-dimensional images [16,17], and their performance is sensitive to illumination conditions, water reflections, and other environmental variations.

Multi-sensor fusion approaches combine image information obtained from vision sensors with three-dimensional information provided by LiDARs or other ranging sensors [18,19,20]. Talha et al. [21] proposed a camera–LiDAR fusion framework that uses calibrated fusion data to compute pothole size and depth, enabling pothole damage severity assessment. Roman-Garay et al. [22] performed semantic segmentation on 2D images and subsequently matched the results with corresponding point clouds for pothole detection. Felix Kortmann et al. [23] adopted an end-to-end concept and constructed two lightweight neural networks for detecting road damage and assessing damage severity, respectively. While such methods can achieve more accurate geometric quantification of potholes, they typically involve complex sensor calibration and synchronization procedures and impose higher requirements on hardware cost and computational resources.

In terms of occupant ride comfort when vehicles traverse uneven road profiles such as depressions or bumps, S. Hegazy et al. [24] investigated body vibration responses and ride-comfort indicators under different road conditions using a classical two-degree-of-freedom quarter-car model. They demonstrated the influence of suspension parameters on vertical acceleration responses, providing a theoretical basis for subsequent comfort-oriented control strategies. Similarly, J. Gao et al. [25] examined the relationship between vehicle speed and passenger vertical comfort when passing over bump roads, and employed a quarter-car model to analyze the effects of different driving speeds and suspension parameters on vertical responses, offering an analytical framework for comfort optimization via speed planning. However, these studies mainly focus on suspension design and control, and provide limited guidance on pothole traversal strategies.

Regarding pothole traversal strategies, Raja et al. [26] proposed a Smart Pothole-Avoidance Strategy (SPAS) for safe vehicle navigation in dense pothole environments, combining passenger feedback and sensor data to determine the timing, speed, and angle for lane changes or speed adjustments. Kuan et al. [27] employed a Deep Q-Network (DQN) as an intelligent agent and conducted cross-task unsupervised transfer learning on the CARLA simulator, enabling pothole detection via vision and subsequent lane-change-based avoidance. Deep learning [28] and end-to-end approaches have also been explored for pothole traversal [29,30,31]. However, most existing studies focus primarily on pothole traversal or braking actions, with relatively simple control strategies and limited consideration of ride comfort during pothole traversal. There is often a lack of a direct and actionable mapping between pothole geometric information and comfort constraints, resulting in insufficient attention to the question of how to convert geometric perception results into executable comfort-oriented vehicle speed planning and longitudinal control commands.

From an engineering perspective, pothole traversal is often unavoidable in real traffic—especially on ordinary urban roads—making it more practical to proactively regulate the vehicle’s longitudinal speed rather than relying solely on lateral avoidance maneuvers. Therefore, this study is positioned as an engineering application-oriented verification framework that bridges pothole identification, vehicle–pothole vertical dynamic analysis, and comfort-constrained longitudinal speed control. Specifically, the proposed method translates perceived pothole geometry into comfort-based limiting speed constraints and executable longitudinal control commands, enabling speed reduction to be completed within a finite preview distance while satisfying both comfort and braking safety requirements.

The main contributions of this study are summarized as follows:

(1) A fusion framework combining a camera and a single-point ranging LiDAR is developed to enable real-time acquisition of pothole contour, area, and depth, achieving a balance between implementation cost and geometric quantification capability.
(2) A vehicle–pothole dynamic model is established in ADAMS, and multiple comparative simulations are conducted to analyze the effects of pothole area and depth on vehicle vertical vibration. The vertical weighted RMS acceleration is introduced as the ride comfort metric, based on which the maximum allowable vehicle speed for traversing potholes under different geometric conditions is determined.
(3) A longitudinal safety-distance model and a fuzzy controller are designed using the distance-to-safety-margin and speed-to-limit-margin as inputs to generate the desired acceleration/deceleration commands. The proposed strategy is validated via MATLAB/Simulink, CarSim co-simulation and further demonstrated through real-vehicle experiments, showing that the vehicle can reduce speed to the allowable limit before pothole traversal and improve ride comfort without compromising braking safety.

**Figure 1:**
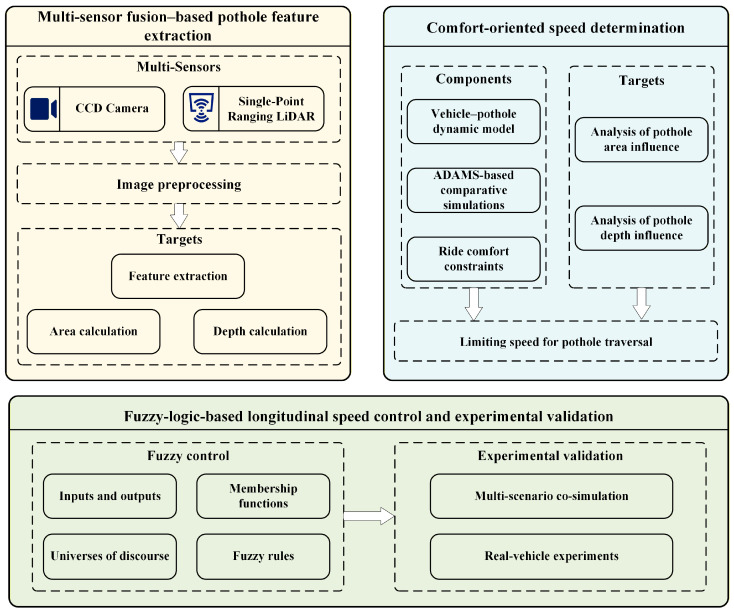
Overall framework of the proposed comfort-oriented pothole traversal method.

The remainder of this paper is organized as follows. Section 2 presents the pothole feature extraction method, including image preprocessing, contour/area computation, and LiDAR-based depth estimation. Section 3 establishes the comfort evaluation framework and derives the limiting traversal speed map based on ADAMS simulations. Section 4 develops a fuzzy-logic-based speed control algorithm for pothole traversal and analyzes multi-scenario co-simulation results on the MATLAB/Simulink–CarSim platform. Section 5 presents real-vehicle experiments to further validate the proposed approach under practical road conditions. Finally, Section 6 concludes the paper and discusses potential directions for future work. The overall structure of the paper is illustrated in Figure 1.

## 2. Pothole Feature Extraction

**Figure 2:**
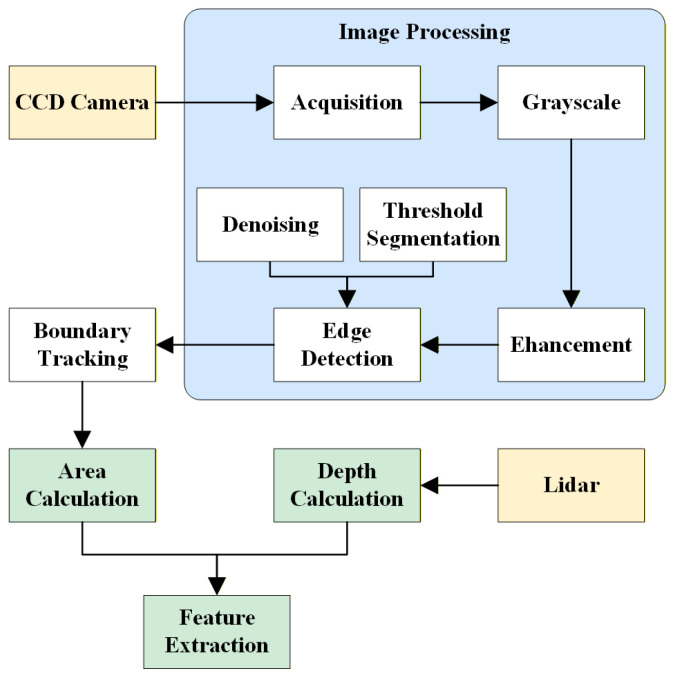
Flowchart of pothole feature-parameter extraction.

Road potholes are the most frequent form of road damage, and the contour, area and depth information of road potholes have an important impact on the safety and comfort of vehicles. In this section, the main features of road potholes are extracted based on a multi-sensor fusion method, including the calculation of pothole contour and area based on visual images and the calculation of pavement pothole depth based on single-point ranging LIDAR. Figure 2 shows the flow chart of pavement pothole feature value calculation.

### 2.1. Image Preprocessing

During the acquisition of road-surface pothole images using a camera, a large amount of noise is inevitably introduced due to variations in lighting conditions, sensor characteristics, and environmental factors, which adversely affect the accuracy of pothole contour extraction. To address this issue, image preprocessing is performed on the captured images in this section, mainly including grayscale conversion and image enhancement.

The road-surface pothole images captured by the CCD camera are color images composed of three information channels, namely red (R), green (G), and blue (B). The grayscale value of each channel ranges from 0 to 255, where a value of 0 indicates black and a value of 255 indicates white. Considering that processing three-channel grayscale information involves a large data volume and high computational cost, a weighted averaging method is adopted in this study. According to the sensitivity of the human visual system to the R, G, and B components, different weighting coefficients are assigned to each channel. This approach enables the conversion of the original color image into a single-channel grayscale image while preserving essential image features, thereby improving processing efficiency and facilitating subsequent image storage and computation. The grayscale conversion formula is given in Equation (1):

(1) P(i,j)=0.299⋅R(i,j)+0.578⋅G(i,j)+0.114⋅B(i,j)

where (i,j) is the coordinates of pixels, P(i,j) is the pixel value of the grayscale image.

In the process of image acquisition, non-uniform illumination conditions and sensor sensitivity can directly lead to the loss of image detail, resulting in locally overexposed or underexposed regions. According to the Retinex color constancy theory, the color perceived by the human visual system is determined by the intrinsic reflectance properties of objects and is independent of illumination conditions. When applied to image processing, Retinex-based methods can preserve the fidelity of the original image while enhancing both dark and bright regions.

In this study, a Multi-Scale Retinex (MSR) algorithm is adopted based on Retinex theory to alleviate the non-uniform illumination effects in pothole images. Its mathematical formulation is expressed as

(2) s(i,j)=l(i,j)r(i,j)

where l(i,j) and r(i,j) represent the incident illumination image and the reflectance image, respectively; s(i,j) denotes the product of the two components.

Taking the logarithm on both sides of Equation (2) yields

(3) log(s(i,j))=log(l(i,j)r(i,j))=log(l(i,j))+log(r(i,j))

Let

(4) $$\left\{\begin{array}{l} S(i,j)=\log(s(i,j))\\ L(i,j)=\log(l(i,j))\\ R(i,j)=\log(r(i,j)) \end{array}\right.$$

then

(5) S(i,j)=L(i,j)+R(i,j)

By convolving a Gaussian function F(i,j) with the incident illumination image, the illumination component of the original image can be estimated. The corresponding mathematical expressions are given by

(6) l(i,j)=F(i,j)∗s(i,j)

(7) $$F(i,j)=\lambda e^{-\frac{i^{2}+j^{2}}{\sigma^{2}}}$$

where ∗ represents the convolution operation; λ is the normalization coefficient; σ is the scale parameter of the Gaussian function.

**Figure 3:**
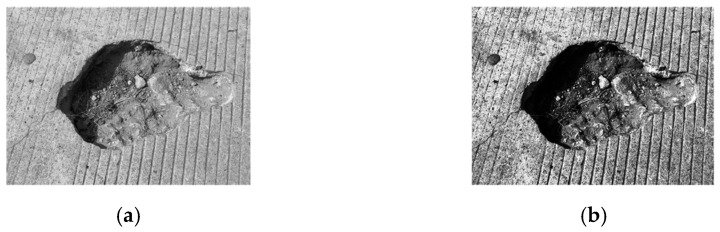
Comparison of image enhancement effect. (**a**) Original grayscale image; (**b**) Enhanced image.

Equation (8) illustrates the principle of the Multi-Scale Retinex (MSR) algorithm. By performing a weighted summation over multiple scales, the MSR method overcomes the limitations of single-scale Retinex algorithms, which may lead to inconsistent preservation of image details at different scales. As a result, the MSR approach achieves both high-fidelity image reconstruction and effective enhancement of local image details. A comparison of the image enhancement results before and after processing is shown in Figure 3.

(8) $$R_{MSR}(i,j)=\sum_{n=1}^{N}W_{n}\left\{\log(s(i,j))-\log[F_{n}(i,j)*s(i,j)]\right\}$$

where n is the number of Gaussian kernels, which is typically set to 3, and $W_{1}=W_{2}=W_{3}=1/3$.

### 2.2. Contour Extraction Based on Threshold-Based Segmentation

**Figure 4:**
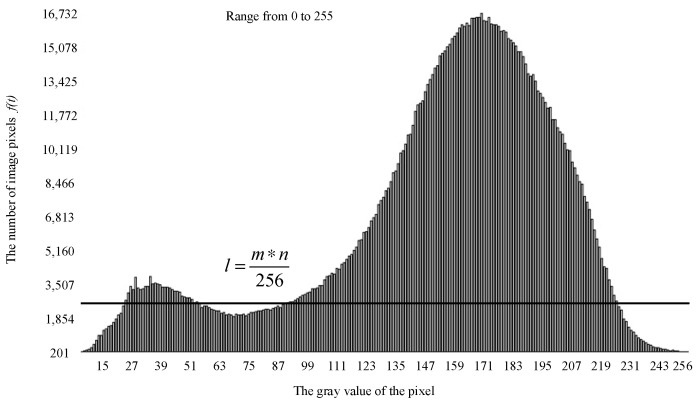
Bimodal gray histogram.

Regarding the characteristics of the grayscale histogram distribution of the preprocessed image, the calcHist function is applied to compute the channel index of the image. The horizontal axis of the histogram represents the gray value of the pixel and the vertical axis represents the number of pixels contained in the gray value. When the grayscale of the target object has a difference from that of the background, the pixel grayscale will be concentrated in two places, showing a double-peak characteristic. From Equation (9), the straight line l is plotted in the grayscale histogram as shown in Figure 4. This line represents the average of pixels of the whole image and necessarily intersects with the two peaks of the histogram to produce four intersection points. The grayscale values of these four intersection points can be calculated to determine the grayscale range where the two peaks are located.

(9) $$l=\frac{m*n}{256}$$

where m and n are the pixel width and height of the image captured by the camera, respectively.

Based on the known gray-level ranges of the two peaks, the min-MaxLoc function is employed to determine, within the left and right peak intervals $t_{l}$ and $t_{r}$, the maximum pixel value and its corresponding gray level for each peak.

To accurately extract road potholes from the images, it is necessary to determine an appropriate image segmentation threshold. First, the initial threshold $T_{0}$ is computed using Equation (10), which divides the image into two parts: the target object and the background. Then, the gray-level means of these two parts, denoted as $t_{l-m}$ and $t_{r-m}$, are calculated using Equations (11) and (12), respectively. The updated threshold $T_{k+1}$ is subsequently obtained according to Equation (13):

(10) $$T_{0}=\frac{t_{l}+t_{r}}{2}$$

(11) $$t_{l-m}=\frac{\sum_{Q(i,j)<T_{k}}Q(i,j)N(i,j)}{\sum_{Q(i,j)<T_{k}}N(i,j)}$$

(12) $$t_{r-m}=\frac{\sum_{Q(i,j)>T_{k}}Q(i,j)N(i,j)}{\sum_{Q(i,j)>T_{k}}N(i,j)}$$

(13) $$T_{k+1}=\frac{t_{l-m}+t_{r-m}}{2}$$

where $t_{l}$ and $t_{r}$ are gray values of the left and right peaks in the histogram, respectively; Q(i,j) is the gray scale of any point in the image; F(i,j) is the number of pixels of pixel point $\left(i,j\right)$; and $T_{k}$ is the threshold.

**Figure 5:**
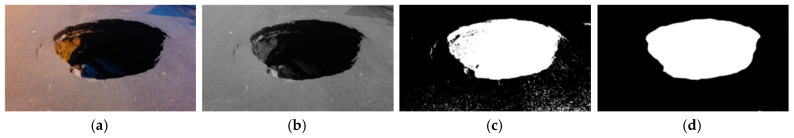
Road pothole edge extraction. (**a**) Original image; (**b**) Grayscale image; (**c**) Binary image; (**d**) Median filtering image.

If the equation $T_{k}=T_{k+1}$ is satisfied, the optimal threshold of image segmentation is attained. Figure 5c shows the binary image after threshold segmentation. The gray value of all pixels in the pothole area is set to be 255 (white), and the gray value of external pixels is set to be 0 (black). As it is simple to calculate and easy to implement, this algorithm can extract the pothole from the pavement background. As shown in Figure 5c, there is still a large amount of salt-and-pepper noise after the above processing. Median filtering is implemented to remove such noise, and the effect is shown in Figure 5d. In addition, the basic therapy of median filtering is that is firstly defines a window of length L, satisfying the relation L=2N+1, and N is a positive integer. The signal in this window is assumed as $f_{i-n},\ldots ,\;f_{i-1},\;f_{i},\;f_{i+1},\ldots ,\;f_{i+n}$ and ordered from small to large. The median $f_{i}$ of the sequence is taken to replace the grayscale value of the specified point (generally the center point of the original window), and the mathematical expression is shown in the following equation.

(14) $$y_{i}=med\left\{f_{i-n},\ldots ,f_{i-1},f_{i},f_{i+1},\ldots ,f_{i+n}\right\}$$

### 2.3. Image-Based Computation of Pothole Area

**Figure 6:**
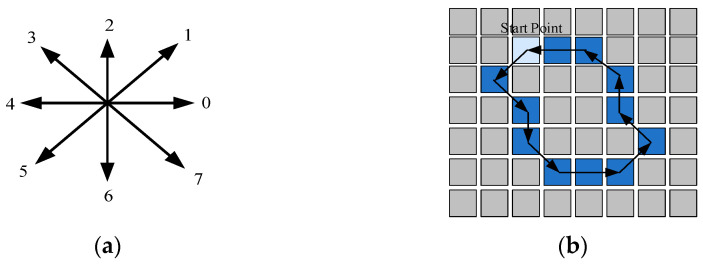
Schematic illustration of the Freeman chain-code representation. (**a**) Eight-direction chain-code map; (**b**) Chain-code curve.

After acquiring the contour features of road potholes, the pixel points at the boundary of the potholes need to be encoded. Freeman code is a methodology of expressing a curve or boundary in terms of the coordinates of the curve’s start point and the direction code of the boundary point. Subsequently, after determining the starting point of the line segment and coding in the counterclockwise direction, the direction of the line is represented by the number in the eight-way Freeman code. As shown in Figure 6b, the Freeman code of the curve is 57670013234.

**Figure 7:**
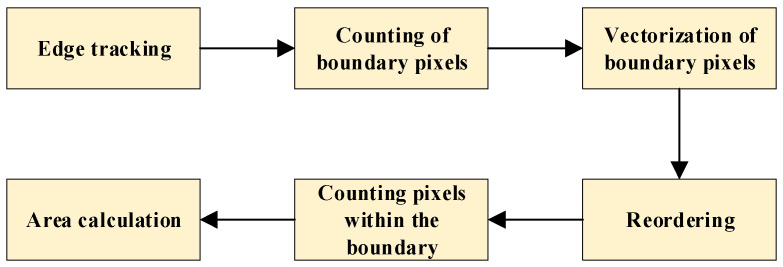
Flowchart of the pothole area calculation algorithm.

In this paper, a similarity-based tracking method for pothole boundaries is developed on the basis of the Freeman chain code, and a corresponding algorithm for calculating pothole area is proposed. The flowchart of the algorithm is shown in Figure 7.

First, the preprocessed image is divided into multiple columns. Starting from the upper-left corner, 8-direction Freeman chain-code tracking is performed along the pothole boundary in a counterclockwise direction and in ascending index order, until the starting point is reached. In this way, an ordered set of boundary-labeled pixels is obtained, and the largest index at the end of the tracking process corresponds to the total number of pothole boundary pixels, denoted as $A_{1}$.

The current boundary pixel is treated as the core boundary point N. The vector from the previous boundary point N−1 to the core point N is defined as the forward vector (fv), and the vector from the core point N to the next boundary point N+1 is defined as the backward vector (bv). Both the vector directions and their corresponding values are encoded using the 8-direction Freeman chain code, with the chain-code center located at the core boundary point N.

To reduce the computational burden, the encoded pothole boundary pixels are reordered in a raster-scan manner, from top to bottom and from left to right. The interior pixels within the pothole boundary are likewise reordered using the same strategy.

If the pixel located to the right of the core boundary point N, i.e., pixel N+1, is classified as an interior point, then the number of pixels between the two boundary pixels in the same row is counted and denoted as $N_{i+1}-N_{i-1}-1$, where $N_{i}$ and $N_{i+1}$ represent the column indices of the corresponding boundary pixels. The total number of pixels inside the pothole boundary, $A_{2}$, is obtained by summing these quantities over all rows. When the decision criterion given in Equation (15) is satisfied, the core boundary point N is identified as an interior boundary point.

(15) $$\left(bv\neq 8\wedge fv=5)\vee (fv<3\wedge \left|fv-bv\right|>4)\vee (fv>5\wedge \left|fv-bv\right|<4\right)$$

Finally, the area of the target road-surface pothole is computed based on the formula as follows:

(16) $$S=\left(\frac{1}{2}A_{1}+A_{2}\right)\times u$$

where $A_{1}$ denotes the total number of boundary pixels of the pothole; $A_{2}$ represents the total number of pixels enclosed by the boundary; and u denotes the pixel equivalent used for converting pixel counts to physical dimensions.

### 2.4. Pothole Depth Estimation Using a Single-Point Ranging LiDAR

For potholes, which are negative obstacles located below the road surface, it is difficult to obtain accurate depth information using only two-dimensional images captured by a camera. LiDAR, however, can provide direct depth measurements of the target. Therefore, in this study, a perception system is constructed by combining a single-point ranging LiDAR with road-surface pothole images acquired by a CCD camera, enabling rapid estimation of both pothole depth and area.

In practical driving conditions, uncertainty may exist in LiDAR-based depth estimation due to vehicle vibration, road surface irregularities, and the possibility that the laser beam does not intersect the deepest point of an irregular pothole. Such uncertainty may introduce deviations in the estimated pothole depth and consequently affect the traversal speed determined in the speed-planning module. In particular, an underestimation of pothole depth may lead to a higher planned traversal speed, whereas an overestimation may result in a more conservative braking response. To mitigate this effect, the LiDAR measurement is aligned with the camera-based pothole region of interest, and a conservative safety margin is incorporated into the speed-planning process. This strategy helps ensure that the resulting control decisions remain safe and comfort-oriented even in the presence of moderate depth estimation errors.

**Figure 8:**
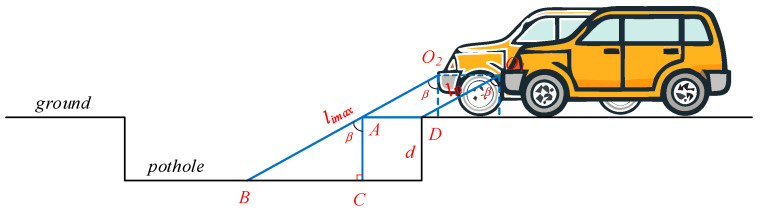
Geometric model of pothole depth measurement using a single-point ranging LiDAR.

The geometric model for LiDAR-based road-surface pothole depth measurement is shown in Figure 8. The single-point LiDAR sensor is rigidly mounted at point O on the front bumper in front of the wheel by means of a bracket and is installed with a downward pitch angle θ. Points $O_{1}$ and $O_{2}$ denote the positions corresponding to the same fixed point O at different stages of the vehicle’s motion. Segment $O_{1}O_{2}$ is a horizontal line, and AD is the virtual extension of the flat road surface. Thus, quadrilateral $O_{1}O_{2}AD$ forms a parallelogram, and $O_{1}D=O_{2}A$.

When the vehicle is traveling on an intact, pothole-free road surface, the distance from the LiDAR measurement point to the road surface is $L_{1}$. Once a pothole appears, the measured distance gradually increases, and at the deepest point of the pothole, the distance becomes $L_{2}$. Both $L_{1}$ and $L_{2}$ are directly obtained from the LiDAR range data. In the right triangle ΔABC, the geometric relationship between these quantities is used to derive the pothole depth d, which can be calculated according to Equation (17).

(17) $$d=AC=cos\beta \cdot AB=cos\beta \cdot (l_{imax}-l_{i0})$$

## 3. Comfort-Constrained Allowable Speed for Pothole Traversal

In this section, the effects of road-surface potholes with different areas and depths on vehicle ride comfort are analyzed using ADAMS(2017) software. The weighted root-mean-square (RMS) acceleration is employed as the vibration impact index, and the recommended vehicle speed for traversing each type of pothole that satisfies human ride-comfort requirements is subsequently determined.

### 3.1. Vehicle–Pothole Dynamic Modeling and Comfort Metric

In this study, a vehicle dynamics model is developed based on the virtual prototype MDI_Demo_Vehicle in Adams/Car. The road model is generated using the Road Builder module, in which the pothole parameters—including area (width and length), depth, and road-surface friction coefficient—are specified.

To enable systematic parametric analysis, the pothole geometry is idealized as a square planform with a uniform depth, so that the effects of area and depth can be examined in a controlled manner. Although real-world potholes may exhibit irregular boundaries and non-uniform depth profiles, this idealization provides an engineering-level approximation for deriving a comfort-constrained limiting-speed map. The potential impact of irregular pothole morphology on the mapping and its external validity is discussed as a limitation, and will be addressed in future work by incorporating more realistic pothole shapes and depth fields.

According to the classification method in [32], potholes are categorized by size as shown in Table 1. In the subsequent simulations, the pothole area is further subdivided with an interval of 0.5 m^2^ to obtain a more accurate estimation of the maximum allowable vehicle speed for pothole traversal.

Besides, in this work, the suspension, damping, and tire models provided by the ADAMS/Car built-in vehicle library are adopted with default parameter settings. This modeling choice is intended to ensure modeling consistency and engineering representativeness, rather than pursuing component-level parameter identification or optimization. Accordingly, the emphasis of this work is on deriving the relative effects and the comfort-constrained limiting-speed mapping with respect to pothole geometry under consistent vehicle dynamics assumptions. It should be noted that the resulting speed map is specific to the considered vehicle configuration and should be recalibrated when applied to vehicles with substantially different wheelbase, suspension travel, or sprung mass.

The dominant discomfort induced by road-surface potholes is associated with vehicle vertical vibration experienced by the occupants. In this work, the weighted root-mean-square (RMS) acceleration is adopted to evaluate the influence of different vehicle speeds and pothole conditions on ride comfort. Since potholes primarily affect the vehicle’s vertical vibration while having a relatively minor influence on longitudinal and lateral vibrations, the vertical weighted RMS acceleration ${\bar{a}}_{w}$ is used as the main evaluation metric. The calculation formula is given as follows:

(18) $${\bar{a}}_{w}={\left[\int_{0.5}^{80}{W_{k}}^{2}(f)G_{a}(f)df\right]}^{\frac{1}{2}}$$

where $G_{a}(f)$ is the acceleration power spectral density, f is the frequency, and ${W_{k}}^{2}(f)$ is the vertical frequency weighting function. The detailed calculation is given as follows:

(19) $$W_{k}\;(f)=\left\{\begin{array}{l} 0.5\;\;(0.5<f<2)\\ f/4\;\;\;\;\;(2<f<4)\\ 1\;\;\;\;\;(4<f<12.5)\\ 12.5/f\;\;(12.5<f<80) \end{array}\right.$$

The relationship between weighted RMS acceleration and subjective passenger perception is summarized in Table 2. In this study, a comfort threshold ${\bar{a}}_{T}=0.315$ is defined for the vertical weighted RMS acceleration. When the vertical weighted RMS acceleration exceeds this threshold, i.e., ${\bar{a}}_{w}>{\bar{a}}_{T}$ the vehicle occupants are considered to experience discomfort. Conversely, if the inequality ${\bar{a}}_{w}\leq {\bar{a}}_{T}$ is satisfied, the vehicle is regarded as not causing noticeable discomfort to the occupants during pothole traversal.

It should be noted that the ISO 2631-based comfort boundary adopted in this study serves as a practical engineering threshold for speed-planning constraint generation. Individual comfort perception may vary with seating position, exposure duration, and human sensitivity; therefore, the comfort threshold can be reconfigured or calibrated for specific user groups or application requirements. In this work, we use the selected threshold to provide a conservative and interpretable criterion for comparing different pothole geometries and traversal speeds under a consistent evaluation protocol.

### 3.2. Parametric Simulation Results and Speed-Limit Mapping

**Figure 9:**
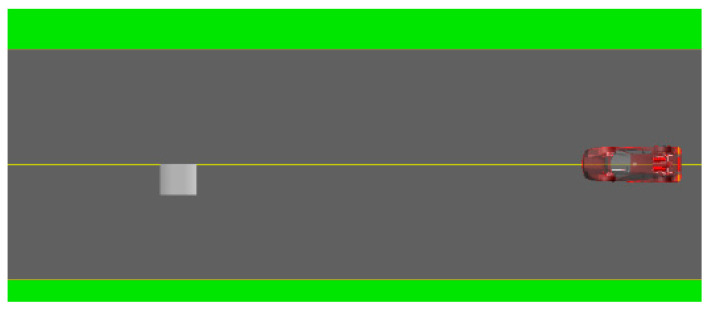
Schematic diagram illustrating the simulation conditions of road potholes.

In the road modeling module, a road profile file is generated using a single-side pothole as the analysis object, with the front wheel designated as the detecting wheel. The pothole geometry is simplified as a square, with its area ranging from 0 to 3 m^2^ and depth ranging from 0 to 0.1 m. The area and depth are incremented by 0.5 m^2^ and 0.03 m, respectively. The vehicle is set to traverse the pothole at constant speeds of 10 km/h, 20 km/h, 30 km/h, 40 km/h, 50 km/h, and 60 km/h. The starting position of the pothole is defined at an X-axis coordinate of −5 m, with the Y- and Z-axis coordinates set to 0 m. The terminal positions along the Y- and Z-axes remain unchanged. At the same time, the simulation duration is 5 s, with a total of 500 simulation steps. The simulation condition is illustrated in Figure 9.

**Figure 10:**
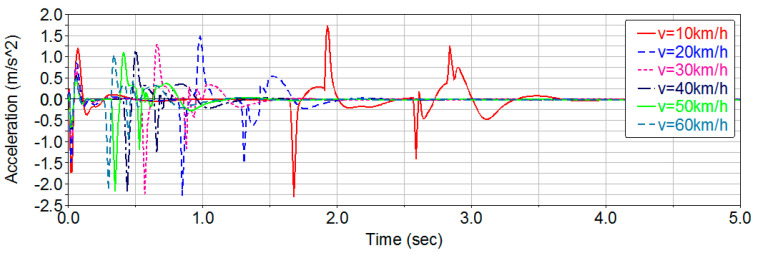
Vertical acceleration of the vehicle at different speeds.

To investigate the effects of pothole size and vehicle speed on ride comfort, multiple simulation scenarios must be configured. First, a road-surface pothole with an area of 0.5 m^2^ and a depth of 0.03 m is generated. The vehicle then traverses the pothole at speeds ranging from 10 to 60 km/h. The resulting vertical acceleration responses of the vehicle are shown in Figure 10.

**Figure 11:**
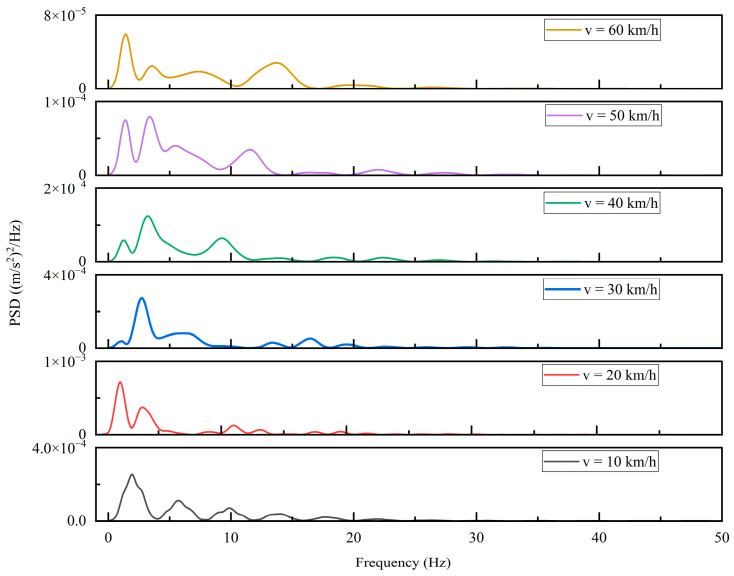
Power spectral density of the vehicle at different speeds.

From the figure above, it can be observed that the vertical vibration is most pronounced when the vehicle speed is 10 km/h. By applying a Hamming window to the vertical acceleration time-domain signal and performing a Fast Fourier Transform (FFT), the corresponding acceleration power spectral density (PSD) curve is obtained, as shown in Figure 11. Based on this PSD, the vertical weighted RMS acceleration can be calculated, the result is ${\bar{a}}_{w}=0.3112\;{m/s}^{2}<{\bar{a}}_{T}$.

Therefore, when the vehicle traverses the pothole at 10 km/h—the speed that produces the largest vertical vibration—the vertical weighted RMS acceleration remains below the human comfort threshold, indicating that the occupants do not experience discomfort. For this pothole geometry (S ≤ 0.5 m^2^, depth < 0.03 m) and the tested speed range (10–60 km/h), the weighted RMS remains below the comfort threshold; therefore, no additional speed reduction is required within this range.

Next, the pothole area is varied to investigate its effect on vehicle ride comfort. Further simulations are conducted for the pothole-area range corresponding to the minor-damage category to examine the influence of pothole size in greater detail. The pothole area is set to 1 m^2^, while the depth is kept at 0.03 m, and all other parameters remain unchanged. The resulting vertical RMS acceleration values of the vehicle at different speeds are summarized in Table 3, where g denotes the gravitational acceleration.

Based on the results in Table 3, when the vehicle speed is 20 km/h, the vertical weighted RMS acceleration ${\bar{a}}_{w}=0.338$ exceeds the human comfort threshold ${\bar{a}}_{T}=0.315$, indicating that occupant comfort cannot be ensured under this condition. In contrast, at speeds of 10, 30, 40, 50, and 60 km/h, the condition ${\bar{a}}_{w}<{\bar{a}}_{T}$ is satisfied, and the ride comfort requirement is met. Therefore, the maximum allowable vehicle speed for traversing this pothole can be determined as $v_{l}\leq 10$ km/h or $v_{l}\geq 30$ km/h (depending on whether the lower or the next feasible speed level is selected as the limiting value).

**Figure 12:**
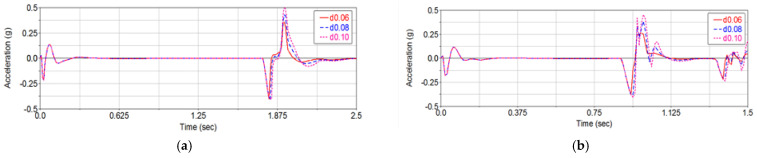

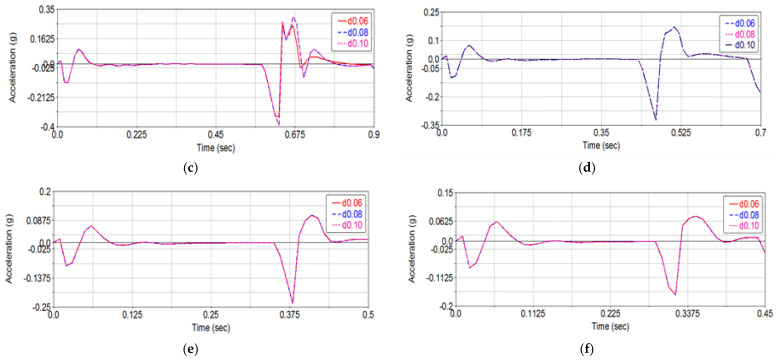
Vertical acceleration of the vehicle at different speeds and pothole depths for a fixed pothole area. (**a**) v=10 km/h; (**b**) v=20 km/h; (**c**) v=30 km/h; (**d**) v=40 km/h; (**e**) v=50 km/h; (**f**) v=60 km/h.

Next, the influence of pothole depth on vehicle ride comfort is examined using the same approach. The pothole area is fixed at S = 1 m^2^, while the depths are set to 0.06, 0.08, and 0.10 m, with all other parameters kept unchanged. Based on multiple simulation runs, the vertical acceleration responses of the vehicle at different speeds for these pothole depths are obtained, as shown in Figure 12.

As shown in Figure 12, when the pothole area is kept constant and the vehicle speed satisfies v≥40 km/h, the vertical acceleration responses of the vehicle when passing over potholes with depths of 0.06, 0.08, and 0.10 m almost coincide. Under the same conditions, the maximum absolute value of the vertical acceleration at a speed of 10 km/h is greater than that at 60 km/h, indicating that a higher vehicle speed actually leads to a smaller vertical vibration response when traversing the pothole.

According to the pothole damage severity classification in Table 1, multiple comparative simulations are conducted for road-surface potholes with areas ranging from 0 to 3 m^2^ and depths ranging from 0 to 0.1 m. Table 4 summarizes the comfort-oriented traversal speed constraints derived from the simulation above. For a fixed vehicle configuration, potholes with different areas and depths are constructed, and the vehicle traverses each pothole at multiple candidate speeds. The vertical acceleration responses are recorded and evaluated using the ISO 2631-based comfort metric; the speeds satisfying the comfort threshold are then identified to form the speed map. Based on these simulations, the recommended vehicle speeds that ensure occupant comfort under different combinations of pothole area and depth are obtained.

## 4. Pothole-Aware Longitudinal Speed Planning and Control

Based on the analyses and investigations presented in the previous sections, the pothole detection method and the limiting vehicle speed that ensures ride comfort during pothole traversal have been obtained. Building upon these results, this section further integrates fuzzy logic theory with vehicle dynamic characteristics to design a longitudinal comfort-oriented pothole traversal algorithm, aiming to enhance driving safety and occupant comfort when vehicles pass over road-surface potholes.

### 4.1. Longitudinal Safety Distance Model

For intelligent vehicles, the required braking deceleration to avoid road-surface potholes and other negative obstacles ahead can be characterized using a safety distance model. When the pothole detection system identifies a pothole on the upcoming road, it is necessary to compute the minimum safe distance between the vehicle and the pothole under the current driving conditions in order to ensure safety and comfort during traversal. When the actual distance between the vehicle and the pothole is less than or equal to the calculated safe distance, the system issues a braking command.

According to the current vehicle speed v and the limiting pothole-traversal speed $v_{l}$ obtained in the previous section, a longitudinal safety distance model considering the influence of road-surface potholes is established as follows:

(20) $$d=\frac{v_{lim}^{2}-v^{2}}{2a_{max}}$$

where d denotes the safe distance, v is the ego-vehicle speed, $v_{lim}$ is the limiting speed for pothole traversal, and $a_{max}$ is the maximum braking deceleration of the vehicle.

### 4.2. Fuzzy-Logic-Based Speed Regulation Algorithm

In complex and dynamic driving environments, it is difficult to describe the state evolution of the controlled system using unified rules and precise mathematical models. Fuzzy control incorporates driver experience into the control strategy in the form of linguistic rules, enabling effective handling of nonlinear and complex control problems with strong robustness. In this study, fuzzy logic theory is employed to achieve reasonable control of vehicle braking deceleration, thereby improving both the safety and ride comfort of the pothole traversal strategy.

The difference between the actual distance s and the safe distance d between the vehicle and the road-surface pothole is defined as $e_{s}$, while the difference between the ego-vehicle speed v and the limiting pothole-traversal speed $v_{lim}$ is defined as $e_{v}$. The variables $e_{s}$ and $e_{v}$ are selected as the two input variables of the fuzzy controller.

(21) $$\left\{\begin{array}{c} e_{s}=s-d\\ e_{v}=v_{lim}-v \end{array}\right.$$

**Figure 13:**
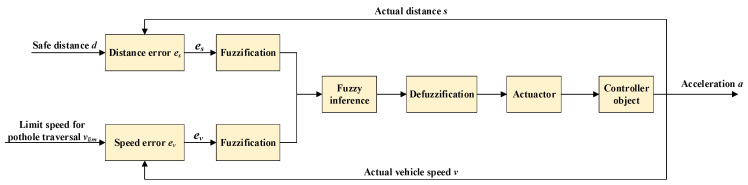
Structure of the fuzzy controller.

Figure 13 illustrates the system structure of the fuzzy controller. The longitudinal pothole traversal algorithm is required to satisfy the following condition: when the distance between the vehicle and the road-surface pothole is greater than zero, the actual vehicle speed must comply with the limiting speed for safe pothole traversal. That is, by adjusting the vehicle acceleration, the fuzzy controller is able to regulate the vehicle speed to within the allowable safe range for pothole traversal over a finite distance, thereby achieving comfortable pothole traversal.

Considering that road maintenance on urban expressways and highways is generally better than that on ordinary urban roads, road surface damage and potholes mainly occur on ordinary roads. Therefore, the simulation scenarios in this study focus on ordinary roads. It is assumed that the maximum vehicle speed on ordinary roads is 80 km/h. Accordingly, the universe of discourse for the input speed difference $e_{v}$ is set to [−80, 80] km/h, where the two extreme values correspond to the most dangerous and the safest driving conditions, respectively.

The camera used in this study has a focal length of 25 mm. Based on the calibrated field-of-view and practical detection performance of our camera setup, the maximum effective preview distance is set to 60 m. The most dangerous condition occurs when the distance difference between the vehicle and the pothole is 0 m. Consequently, the universe of discourse for $e_{s}$ is defined as [0, 60] m. The universe of discourse for the output acceleration a is set to [−10, 10] m/s^2^. Both the input and output variables are described using seven fuzzy linguistic terms: {NB (Negative Big), NM (Negative Medium), NS (Negative Small), ZO (Zero), PS (Positive Big), PM (Positive Medium), PB (Positive Big)}.

For the selection of membership functions, in order to improve the robustness of the fuzzy control algorithm, the input and output membership functions are required to support smooth and continuous fuzzy operations. Gaussian membership functions are adopted for the input variables $e_{s}$ and $e_{v}$, while a combination of Gaussian and triangular membership functions is used for the output variable a. Here, $f\left(e_{v}\right)$, $f\left(e_{s}\right)$, and $f\left(a\right)$ denote the membership functions of $e_{s}$, $e_{v}$ and a, respectively.

Before constructing the fuzzy rules, the fuzzy control logic under the following three typical driving conditions must be clarified:

(1) When $e_{s}$ is small and $e_{v}$ is large, that is, the vehicle is very close to the pothole and the current speed has not yet been reduced to the limiting speed for safe pothole traversal, the vehicle is in a highly dangerous state. In this case, a large braking deceleration should be applied to enhance driving safety over the pothole.

(2) When $e_{s}$ is large and $e_{v}$ is small, meaning that the difference between the actual distance and the safe distance is large and the current vehicle speed is already close to the limiting speed, the vehicle is in a relatively safe state and can maintain its current speed.

(3) When both $e_{s}$ and $e_{v}$ are large or both are small, the braking intensity should be gradually increased so that the vehicle enters a safe state in advance.

For completeness and reproducibility, the 7 × 7 fuzzy rule base (49 rules) used in this study is summarized in Table 5. The rules are constructed to increase braking intensity as the safety margin decreases and/or the speed margin increases, ensuring a generally monotonic risk-to-deceleration mapping with saturation to avoid aggressive commands.

**Figure 14:**
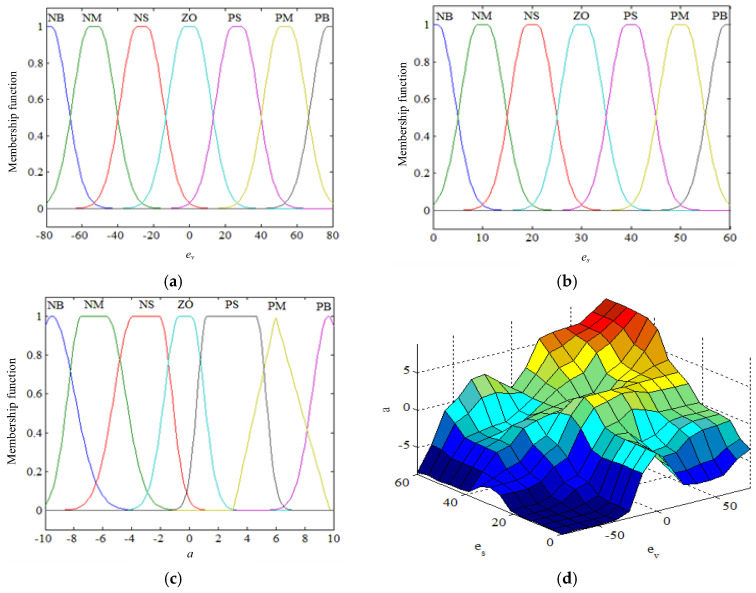
Membership functions and corresponding fuzzy rules. (**a**) Membership functions of $e_{v}$; (**b**) Membership functions of $e_{s}$; (**c**) Membership functions of a; (**d**) Three-dimensional surface of fuzzy control rules.

The membership functions of the input and output variables and the fuzzy control rule surface are shown in Figure 14.

### 4.3. Co-Simulation Setup and Results

To verify the effectiveness and control performance of the proposed algorithm, co-simulation experiments are conducted using MATLAB/Simulink (R2021a) and CarSim (2019.0). Three different road-surface pothole conditions are considered. The road adhesion coefficient is set to 0.85, and the simulation duration is 5 s.

Fuzzy control can better emulate the decision-making characteristics of human drivers and provides a more flexible response to the interaction between vehicle speed and road pothole conditions. To evaluate the effectiveness of the proposed method, a conventional PID controller is introduced as a baseline for comparison in the simulations. In the figure, the red curve denotes the results of the PID controller, whereas the blue curve represents the results obtained using the fuzzy control strategy.

**Figure 15:**
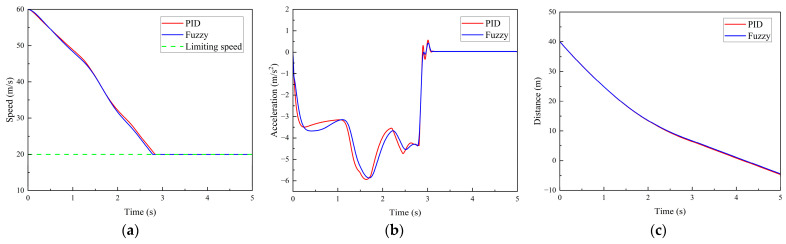
Simulation results for Scenario 1. (**a**) Vehicle speed. (**b**) Vehicle acceleration. (**c**) Distance between the vehicle and the road-surface pothole ahead.

(1) Scenario 1: The vehicle travels on an ordinary road with an initial speed of 60 km/h. A road-surface pothole with an area of 2.7 m^2^ and a depth of 0.03 m is located 40 m ahead of the vehicle’s initial position. After the pothole is detected, the designed comfort-oriented pothole traversal system controls the vehicle based on the pothole information and the ego-vehicle state. The time histories of vehicle speed, acceleration, and the distance between the vehicle and the pothole are shown in Figure 15.

As indicated by the vehicle acceleration curve in Figure 15b, during the time interval from 0 to 1 s, the braking deceleration remains within the range of 3–4 m/s^2^, while the vehicle speed decreases from 60 km/h to 48 km/h, as shown in Figure 15a. Subsequently, the braking deceleration gradually increases and reaches approximately 6 m/s^2^ at around 1.6 s, with the vehicle speed reduced to 37 km/h.

Thereafter, the braking deceleration begins to decrease. At 2.8 s, the vehicle speed drops to 20 km/h, at which point the calculated vertical frequency-weighted RMS acceleration ${\bar{a}}_{w}=0.310<{\bar{a}}_{T}$ is below the comfort threshold, satisfying the ride-comfort requirement. As shown in Figure 15b, under the same simulation condition, the maximum braking deceleration occurs at 1.60 s and 1.73 s under PID control and fuzzy control, respectively, with peak values of −5.92 m/s^2^ and −5.85 m/s^2^. The deceleration fluctuations between 2.9 s and 3.1 s further indicate that the fuzzy control strategy provides a smoother deceleration response than the conventional PID controller.

As shown in Figure 15c, the distance between the vehicle and the pothole remains greater than 0 m throughout the process, indicating that the vehicle can regulate its speed to within the allowable limiting speed for pothole traversal within a finite distance. These results demonstrate that the proposed algorithm satisfies the design requirements.

**Figure 16:**
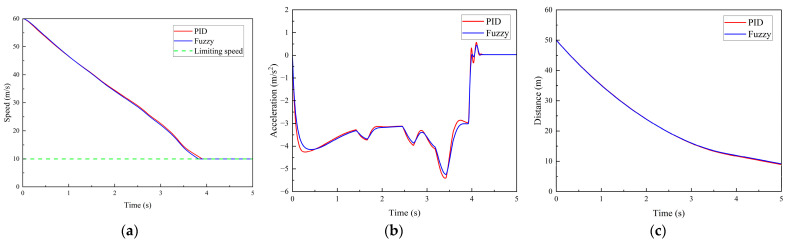
Simulation results for Scenario 2. (**a**) Vehicle speed. (**b**) Vehicle acceleration. (**c**) Distance between the vehicle and the road-surface pothole ahead.

(2) Scenario 2: The vehicle travels normally on the road with an initial speed of 60 km/h. A road-surface pothole with an area of 1.5 m^2^ and a depth of 0.03 m is located 50 m ahead of the vehicle’s initial position. The time histories of vehicle speed, acceleration, and the distance between the vehicle and the pothole are shown in Figure 16.

As shown in Figure 16a, the vehicle speed decreases from the initial 60 km/h to the limiting pothole-traversal speed of 10 km/h, at which the vertical weighted RMS acceleration ${\bar{a}}_{w-k}=0.272<{\bar{a}}_{T}$ is lower than the comfort threshold, satisfying the ride comfort requirement. As illustrated in Figure 15b, during the time interval from 0 to 3.0 s, the braking deceleration remains stable within the range of 3–4 m/s^2^. At approximately 3.4 s, the braking deceleration reaches its peak value of −5.4 m/s^2^ under PID control and −5.25 m/s^2^ under fuzzy control, after which it gradually decreases. By around 4.2 s, the vehicle acceleration approaches 0 m/s^2^.

And from Figure 16c, the distance between the vehicle and the road-surface pothole remains greater than 0 m throughout the maneuver, indicating that the vehicle is able to regulate its speed to within the allowable limiting speed for pothole traversal over a finite distance. The entire control process satisfies the ride comfort requirement during pothole traversal and is also consistent with the driver’s braking decision-making behavior.

**Figure 17:**
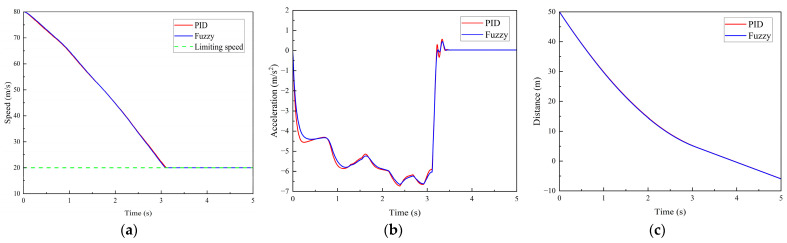
Simulation results for Scenario 3. (**a**) Vehicle speed. (**b**) Vehicle acceleration. (**c**) Distance between the vehicle and the road-surface pothole ahead.

(3) Scenario 3: The vehicle travels on the road with an initial speed of 80 km/h. A road-surface pothole with an area of 0.7 m^2^ and a depth of 0.07 m is located 50 m ahead of the vehicle’s initial position. The time histories of vehicle speed, acceleration, and the distance between the vehicle and the pothole are shown in Figure 17.

During the time interval from 0 to 0.8 s, the braking deceleration reaches 4–5 m/s^2^, corresponding to a reduction in vehicle speed from 80 km/h to 68 km/h, as shown in the speed profile. Subsequently, the braking deceleration further increases to 5–6 m/s^2^, and the vehicle speed decreases to 40 km/h; however, the speed has not yet reached the limiting speed required for safe pothole traversal. When the braking deceleration increases to 6–7 m/s^2^, the vehicle speed is reduced to 20 km/h at approximately 3.0 s. At this moment, the control system calculates that the vertical weighted RMS acceleration ${\bar{a}}_{w}=0.223<{\bar{a}}_{T}$, remains within the comfort threshold.

As can be observed from Figure 17c, the distance between the vehicle and the road-surface pothole is still greater than 0 m at this time, indicating that the proposed pothole traversal control algorithm is capable of regulating the vehicle speed to within the limiting speed before pothole traversal. This ensures driving safety while maintaining satisfactory ride comfort.

From the results of the three simulation scenarios, it can be observed that although both the PID and fuzzy controllers can reduce the vehicle speed to the desired pothole-traversal speed before reaching the pothole, the fuzzy control strategy generates a smoother deceleration response, thereby improving ride comfort during the braking process.

## 5. Real-Vehicle Experiments

This chapter focuses on the real-vehicle validation of the proposed traversal control algorithm for road-surface potholes. A real-vehicle experimental platform is established, and single-pothole road tests are conducted within the constraints of the available experimental equipment to verify the feasibility and control performance of the proposed algorithm on an actual vehicle.

### 5.1. Experimental Platform and Sensors

**Figure 18:**
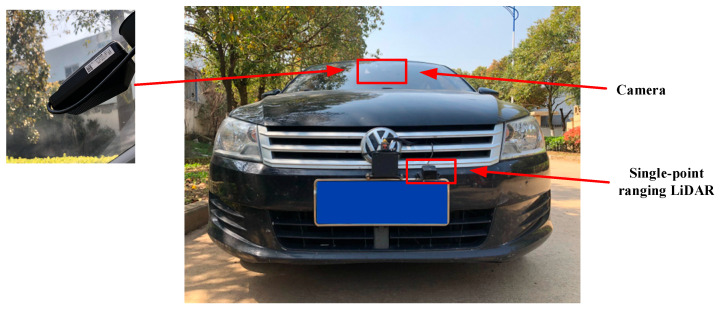
Experimental vehicle.

**Figure 19:**
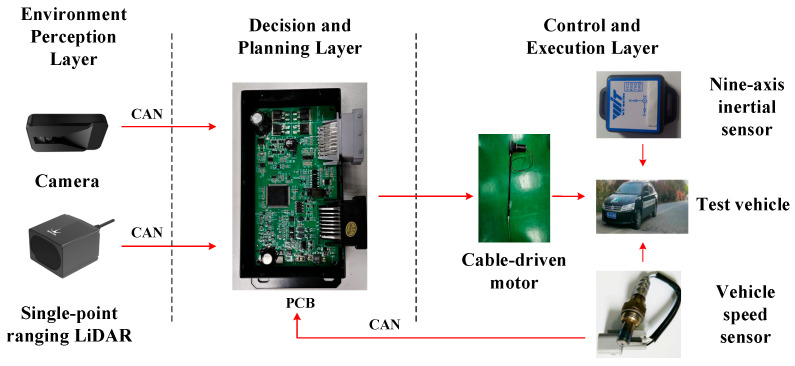
Architecture of the intelligent control system.

A modified vehicle equipped with Level-2 (L2) advanced driver-assistance capabilities is selected as the experimental platform. The exterior view of the vehicle and the sensor layout are shown in Figure 18. To enable online perception of road-surface potholes, a single-point ranging LiDAR and a forward-looking camera are installed on the test vehicle, forming a vision–range fusion–based environment perception system. The overall architecture of the intelligent vehicle control system is illustrated in Figure 19, which mainly consists of the environment perception layer, the decision and planning layer, and the control and execution layer.

In the environment perception layer, the camera and the single-point ranging LiDAR collaboratively acquire key geometric features of road-surface potholes, including pothole area and depth, and provide input information for the subsequent control strategy. The decision and planning layer implements the proposed control algorithm on a PCB-based controller to enable online decision-making and computation. For different pothole areas and depths, the limiting vehicle speed that satisfies human comfort constraints is first determined. Subsequently, based on the deviations between the limiting speed and the current vehicle speed, as well as between the actual distance to the pothole and the safe distance, the required longitudinal acceleration command is calculated.

In the control and execution layer, a cable-driven motor actuates the brake pedal by driving different opening angles according to the pulse voltage output from the controller, thereby regulating the braking input and generating the corresponding braking torque to ensure that the vehicle speed meets the control objectives. For experimental data acquisition and evaluation, acceleration sensors are used to record the longitudinal deceleration during the braking phase and the vertical acceleration signals during pothole traversal, providing the basis for subsequent comparison between the weighted RMS acceleration and the comfort threshold.

### 5.2. Experimental Results and Discussion

To verify the effectiveness of the proposed pothole-detection-based pothole traversal control algorithm under real-road conditions, real-vehicle experiments are conducted for a single road-surface pothole scenario. The experimental conditions are configured as follows. The vehicle travels at a constant initial speed of 35 km/h on a dry, level asphalt road, with the road adhesion coefficient set to 0.85. A single pothole is placed 25 m ahead of the vehicle’s initial position, with an area of 2.8 m^2^ and a depth of 0.03 m. During the experiment, the onboard camera and the single-point ranging LiDAR acquire the pothole depth and area information online. Based on this information, the control algorithm computes the limiting vehicle speed that satisfies the ride-comfort constraints, and the controller outputs longitudinal acceleration/braking commands to drive the actuators and regulate the vehicle speed.

**Figure 20:**
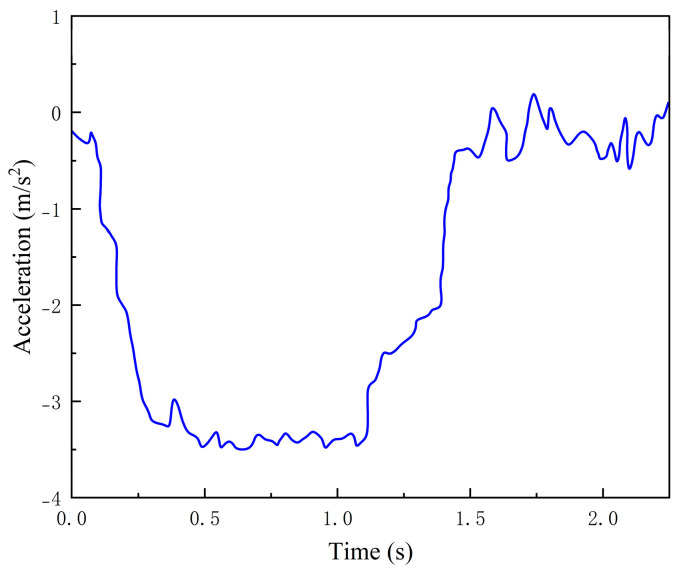
Vehicle acceleration profile.

**Figure 21:**
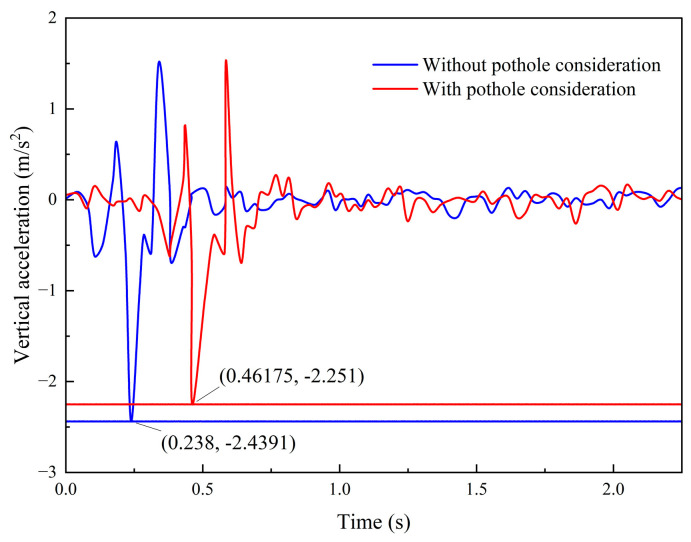
Vehicle vertical acceleration profile.

To ensure a fair comparison, experiments are conducted using the same test vehicle under two modes: with the pothole traversal strategy enabled and without the pothole traversal strategy enabled. The vehicle longitudinal acceleration and the vertical acceleration during pothole traversal are recorded. The experimental results are shown in Figure 20 and Figure 21.

As shown in Figure 20, after the vehicle enters the braking phase, the longitudinal deceleration is recorded in real time by the acceleration sensor. The average braking deceleration throughout the entire braking process is approximately 3.5 m/s^2^, and the deceleration variation is smooth, indicating that the longitudinal speed control exhibits good ride comfort. When the vehicle speed is reduced to the limiting pothole-traversal speed of 20 km/h calculated by the algorithm, the measured distance between the vehicle and the pothole is 14.12 m. This satisfies the safety-distance constraint specified by the control strategy and ensures that the necessary speed adjustment is completed before the vehicle enters the pothole region.

Figure 21 compares the vertical acceleration responses of the vehicle during pothole traversal under the two modes, namely with pothole consideration and without pothole consideration. When the comfort-oriented pothole traversal algorithm is not enabled, the vehicle passes over the pothole at the current speed, resulting in a relatively large vertical impact response. In this case, the vertical acceleration is $\left|a_{z-\max}\right|=2.44\;{m/s}^{2}$, and the vertical weighted RMS acceleration is ${\bar{a}}_{w}=0.319>{\bar{a}}_{T}$, which exceeds the comfort threshold. In contrast, when the traversal strategy is enabled, the vehicle traverses the pothole at the limiting speed, and the vertical response is effectively suppressed. The peak vertical acceleration is $\left|a_{z-\max}\right|=2.25\;{m/s}^{2}$, and the vertical weighted RMS acceleration is ${\bar{a}}_{w}=0.305<{\bar{a}}_{T}$, both of which satisfy the comfort threshold constraint. Compared with the uncontrolled case, the maximum vertical acceleration is reduced by 7.79%, and the vertical weighted RMS acceleration is reduced by 4.39%.

Notably, because ride comfort correlates more strongly with frequency-weighted vibration than with peak acceleration, the reduction in weighted RMS provides a more representative indication of comfort improvement. These results further demonstrate the effectiveness of the proposed comfort-oriented pothole traversal algorithm, which is capable of improving ride comfort during pothole traversal while ensuring safe braking performance under given road adhesion conditions.

## 6. Conclusions

This study addresses the degradation of vehicle safety and ride comfort caused by road-surface pothole damage and proposes a comfort-oriented pothole traversal algorithm tailored for pothole scenarios. First, a multi-sensor fusion perception framework combining a camera and a single-point ranging LiDAR is established to enable online extraction of key pothole features, including contour, area, and depth. Second, a vehicle–pothole dynamic simulation model is developed based on ADAMS, and systematic comparative simulations are conducted to evaluate the effects of pothole area and depth on ride comfort. The vertical weighted root-mean-square (RMS) acceleration is adopted as the comfort evaluation metric, based on which a method for determining the limiting vehicle speed for pothole traversal under different pothole geometries is proposed, providing constraints for speed planning.

Furthermore, a longitudinal pothole traversal control strategy is designed using fuzzy logic theory and validated through MATLAB/Simulink–CarSim co-simulation as well as real-vehicle experiments. The results demonstrate that the proposed method can effectively regulate the vehicle’s longitudinal speed within a given preview distance, ensuring that the vehicle satisfies both the limiting speed for pothole traversal and the comfort threshold constraints. Consequently, the proposed approach enhances ride comfort while maintaining driving safety when traversing road-surface potholes.

Despite the above works, several limitations should be acknowledged. The limiting-speed constraints are derived under a specific vehicle configuration and road adhesion condition, and the parameters may require recalibration for different vehicle types or friction levels. In addition, the perception accuracy may degrade under challenging conditions (poor illumination or water-filled potholes), and the current strategy mainly targets isolated potholes rather than multiple consecutive defects. Future work will focus on enhancing robustness and generality by further extending the proposed framework for broader and more practical real-world applications.

## Figures and Tables

**Table 1:** Classification of road-surface pothole damage severity.

Pothole Severity	Area (m^2^)	Depth (m)
Minor	<1	<0.03
Moderate	1~3	0.03~0.06
Relatively severe	1~3	0.06~0.10
Severe	>3	>0.10

**Table 2:** Relationship between weighted RMS acceleration and human subjective perception.

Weighted RMS Acceleration (m/s^2^)	Subjective Perception
<0.315	Not uncomfortable
0.315~0.63	A little discomfort
0.5~1.0	Fairly discomfort
0.8~1.6	Uncomfortable
1.25~2.5	Very uncomfortable
>2.0	Extremely uncomfortable

**Table 3:** RMS of vertical weighted acceleration at different velocities.

Vehicle Speed v (km/h)	Maximum Absolute Value of Vertical Acceleration $a_{z-\max}$ (g)	RMS of Vertical Weighted Acceleration ${\bar{a}}_{w}$ (m/s^2^)
10	0.2363	0.261
20	0.2261	0.338
30	0.2147	0.229
40	0.2181	0.177
50	0.2160	0.129
60	0.2373	0.274

**Table 4:** Recommended vehicle speed through potholes.

Area s (m^2^)	Depth d (m)	Limit Speed $v_{l}$ (km/h)
0 < s ≤ 0.5	0<d≤0.03	original velocity
	$\begin{array}{l} 0.03<d\leq 0.06 \end{array}$	$v_{l}\geq 50$
	0.06<d≤0.10	$v_{l}\geq 60$
0.5 < s ≤ 1.0	0<d≤0.03	$v_{l}\leq 10$ $\;\mathrm{or}\;v_{l}\geq 30$
	0.03<d≤0.06	$v_{l}\geq 40$
	0.06<d≤0.10	$v_{l}\geq 50$
1.0 < s ≤ 2.5	0<d≤0.03	$v_{l}\leq 10$ $\;\mathrm{or}\;v_{l}\geq 30$
	0.03<d≤0.06	$v_{l}\geq 50$
	0.06<d≤0.10	$v_{l}\geq 60$
2.5 < s ≤ 3.0	0<d≤0.03	$v_{l}\leq 20$ $\;\mathrm{or}\;v_{l}\geq 40$
	0.03<d≤0.06	$v_{l}\geq 50$
	0.06<d≤0.10	unable

**Table 5:** Fuzzy control rule.

a	$e_{s}$							
		NB	NM	NS	ZO	PS	PM	PB
$e_{v}$	NB	NB	NB	NB	NM	NB	NB	NB
	NM	NB	NB	NB	NM	NS	NS	ZO
	NS	NB	NB	NM	NS	ZO	NS	PS
	ZO	NB	NM	NS	ZO	PS	PS	PS
	PS	NM	NS	NS	PS	PS	PS	PM
	PM	NM	ZO	ZO	PS	PS	PM	PM
	PB	NS	ZO	ZO	PS	PM	PB	PB

## Data Availability

The data are not publicly available due to privacy and safety restrictions (e.g., raw videos/point clouds may contain identifiable information).
